# Correlation of multiple endpoints in the first‐line chemotherapy of advanced gastric cancer: Pooled analysis of individual patient data from Japanese Phase III trials

**DOI:** 10.1002/cam4.6818

**Published:** 2023-12-23

**Authors:** Hiroyuki Arai, Madoka Takeuchi, Wataru Ichikawa, Kohei Shitara, Yu Sunakawa, Koji Oba, Wasaburo Koizumi, Yuh Sakata, Hiroshi Furukawa, Yasuhide Yamada, Masahiro Takeuchi, Masashi Fujii

**Affiliations:** ^1^ Department of Clinical Oncology St. Marianna University School of Medicine Kawasaki Japan; ^2^ Graduate School of Mathematical Sciences The University of Tokyo Tokyo Japan; ^3^ Division of Medical Oncology Showa University Fujigaoka Hospital Yokohama Japan; ^4^ Department of Gastrointestinal Oncology National Cancer Center Hospital East Kashiwa Japan; ^5^ Department of Biostatistics, School of Public Health, Graduate School of Medicine The University of Tokyo Tokyo Japan; ^6^ Department of Gastroenterology Kitasato University School of Medicine Sagamihara Japan; ^7^ Department of Internal Medicine Misawa City Hospital Misawa Japan; ^8^ Department of Surgery Kindai University Faculty of Medicine Osaka‐Sayama Japan; ^9^ Comprehensive Cancer Center, National Center for Global Health and Medicine Tokyo Japan; ^10^ Department of Clinical Medicine, School of Pharmacy Kitasato University Tokyo Japan; ^11^ Department of Digestive Surgery Nihon University Itabashi Hospital Tokyo Japan

**Keywords:** advanced gastric cancer, first‐line chemotherapy, postprogression survival, surrogate endpoint

## Abstract

**Background:**

Individual‐level surrogates are important for management in patients treated for advanced gastric cancer (AGC). This study aimed to comprehensively investigate the correlation of multiple clinical endpoints in the first‐line chemotherapy of AGC.

**Methods:**

Individual patient data (IPD) were collected from four Japanese Phase III trials comparing S‐1‐based first‐line chemotherapies (SPIRITS, START, GC0301/TOP‐002, and G‐SOX trials). Patients without Response Evaluation Criteria in Solid Tumors (RECIST)‐based radiological assessments were excluded. Spearman's rank correlation coefficient was tested for correlation among overall survival (OS), progression‐free survival (PFS), and postprogression survival (PPS). OS, PFS, and PPS were compared between responders (best response: complete response or partial response) and nonresponders (best response: stable disease or progressive disease).

**Results:**

The study included a total of 1492 patients. Eighty percent of the patients (*n* = 1190) received subsequent chemotherapies after the failure of each trial's treatment protocol. PFS moderately correlated with OS (Spearman correlation coefficient = 0.66, *p* < 0.005), whereas the correlation between PPS and OS was strong (Spearman correlation coefficient = 0.87, *p* < 0.005). Responders had significantly longer OS (median, 17.7 vs. 9.1 months, *p* < 0.005), PFS (median, 6.9 vs. 2.8 months, *p* < 0.005), and PPS (median, 10.5 vs. 6.0 months, *p* < 0.005) than nonresponders.

**Conclusions:**

Our results reacknowledged the mild surrogacy of PFS and importance of postprogression treatments in patients with AGC receiving first‐line chemotherapy. Consistent longer survival outcomes in better RECIST categories suggested that tumor response might be a useful individual‐level surrogate.

## 1 INTRODUCTION

Gastric cancer (GC) is the fifth most common cancer and the fourth leading cause of cancer‐related deaths worldwide.^1^ Despite recent developments in systemic chemotherapy, advanced gastric cancer (AGC) remains uncurable and leads to an extremely short survival period.^2^ The current global standard of the first‐line treatment of AGC consists of fluoropyrimidine plus platinum compound combined with immune checkpoint inhibitor if the patient has human epidermal growth factor receptor 2 (HER‐2)‐negative GC or with trastuzumab if HER‐2‐positive GC.^3^, ^4^ After the failure of the first‐line treatment, several subsequent chemotherapies, which include paclitaxel plus ramucirumab, irinotecan, nivolumab, trifluridine/tipiracil, and trastuzumab deruxtecan, are available in practice.^5^, ^6^, ^7^, ^8^, ^9^ To achieve the maximum benefit of chemotherapy in AGC, careful management during consecutive treatments is important.^10^

In AGC treatment, overall survival (OS) is usually considered the most clinically meaningful endpoint. However, more optimal short‐term surrogate endpoints are still investigated because of the prolonged follow‐up period for OS. In general, two approaches for assessing surrogate endpoints are important for clinical implementation.^11^ The first type is an individual‐level (patient‐level) surrogate that is associated with the true endpoint in each patient. This surrogate type would be informative for the management of each patient to better predict prognosis. The second type is at the trial level (treatment level), which predicts the effect of treatment on the true endpoints when compared between treatment arms. This surrogate type could shorten the duration of drug development, further leading to the reduction of sample size and ultimately the costs of clinical trials.

In 2013, Paoletti conducted a large‐scale individual patient data (IPD)‐based meta‐analysis, which showed a high correlation of progression‐free survival (PFS) and OS at the individual level but only a modest correlation at the trial level in the first‐line treatment of AGC.^12^ Therefore, despite predicting patient prognosis, the current consensus is that PFS is not an acceptable endpoint, replacing OS in Phase III trials. Other clinical endpoints have not yet been well explored as surrogate endpoints in patients with AGC receiving first‐line chemotherapy.

The increasing availability of active subsequent chemotherapies improves postprogression survival (PPS) in patients with AGC.^13^ A study reported that PPS is strongly associated with OS in patients with AGC treated with first‐line chemotherapy.^14^ However, the effect of responsiveness to the first‐line chemotherapy on PPS remains unclear. Better understanding of clinical outcomes relating to PPS may be useful for the consecutive care of patients with AGC.

Thus, this study aimed to investigate individual‐level correlation of multiple clinical endpoints in the first‐line chemotherapy of AGC using IPD from four Japanese Phase III trials.

## 2 MATERIALS AND METHODS

### 2.1 Patient population and study design

The study population included patients with AGC who enrolled in four randomized Phase III trials of first‐line chemotherapy conducted in Japan: SPIRITS (NCT00150670; enrollment period, between March 2002 and November 2004), START (NCT00287768; enrollment period, between September 2005 and September 2008), GC0301/TOP‐002 (JapicCTI‐050083; enrollment period, between June 2004 and November 2005), and G‐SOX (JapicCTI‐101,021; enrollment period, between January 2010 and October 2011).^15^, ^16^, ^17^, ^18^ As all trials were conducted in the era where trastuzumab had not been introduced in the treatment of HER2‐positive AGC, HER2 status was not assessed in each trial. The SPIRITS trial included patients who received either S‐1 plus cisplatin or S‐1 alone. In the START trial, patients received either S‐1 plus docetaxel or S‐1 alone. In the GC0301/TOP‐002 trial, patients received either S‐1 plus irinotecan or S‐1 alone. In the G‐SOX trial, patients received either S‐1 plus oxaliplatin or S‐1 plus cisplatin. The interval of CT scan for Response Evaluation Criteria in Solid Tumors (RECIST) assessment as defined in the protocol of each trial is shown in Table S1. IPD were collected in collaboration with principal investigators and sponsors (Taiho Pharmaceutical Co., Ltd, Yakult Honsha Co., Ltd, and Daiichi Sankyo Co., Ltd.) of each trial group. Patients are not available for the RECIST‐based radiological assessments were excluded. All clinical trials were approved by the respective competent ethical committees. All patients provided written informed consent before trial enrollment.

### 2.2 Definition of clinical endpoints

OS was defined as the time from randomization to death or to the last documented follow‐up. PFS was defined as the time from randomization to disease progression or death, whichever occurred first, or to the last documented follow‐up. PPS was defined as the interval from progression of first‐line chemotherapy to death or the last documented follow‐up. Tumor response was determined by independent review committees according to RECIST version 1.0: complete response (CR), partial response (PR), stable disease (SD), and progressive disease (PD). Responders were defined as patients with CR or PR and nonresponders as those with SD or PD.

### 2.3 Statistical analysis

Associations among OS, PFS, and PPS were assessed by Spearman's rank correlation coefficient with an interpretation as follows: 0.80–1.00, strong; 0.60–0.79, moderate; 0.40–0.59, fair; 0.00–0.39, weak. The median OS, PFS, and PPS periods were estimated using the Kaplan–Meier method. To compare the survival outcomes between RECIST categories, the log‐rank test was performed. Demographics of the responders and nonresponders were compared using the chi‐squared test for categorical variables and the Mann–Whitney *U*‐test for continuous variables. Statistical analyses were performed using STATA ver.17.0 (Stata Corp., College Station, TX, USA). All tests were two‐sided, and *p* < 0.05 were considered statistically significant.

## 3 RESULTS

### 3.1 Patient and study characteristics

**FIGURE 1:**
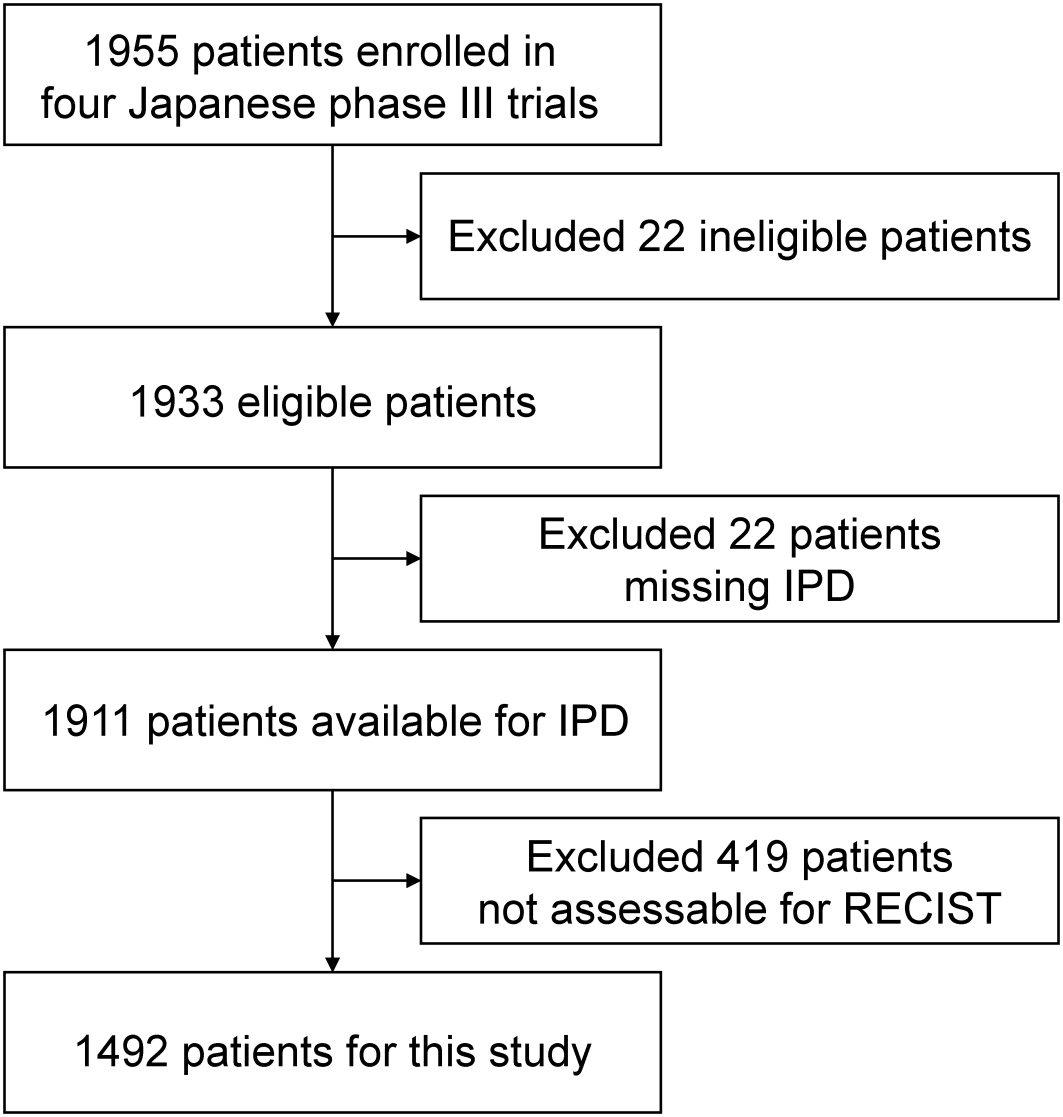
CONSORT diagram. IPD, individual patient data; RECIST, Response Evaluation Criteria in Solid Tumors.

A total of 1955 patients were enrolled in the four trials, and IPD were obtained from 1911 patients. After excluding 419 patients who did not have RECIST assessment, 1492 were included in this study (Figure 1). Male sex, Eastern Cooperative Oncology Group performance status (ECOG PS) 0, presence of primary lesion, and advanced disease at diagnosis were predominant characteristics of the cohort (Table 1).

**TABLE 1:** Patient characteristics.

	Spirits *N* = 185	Start *N* = 474	GC0301/TOP‐002 *N* = 196	G‐SOX *N* = 637	All *N* = 1492
Sex					
Male	145 (78%)	346 (73%)	146 (74%)	474 (74%)	1111 (74%)
Female	40 (22%)	128 (27%)	50 (26%)	163 (26%)	381 (26%)
Age					
Median (Range)	63 (35–74)	65 (23–79)	64 (30–75)	65 (21–85)	65 (21–85)
ECOG PS					
0	134 (72%)	224 (47%)	136 (69%)	452 (71%)	946 (63%)
1	46 (25%)	250 (53%)	51 (26%)	177 (28%)	524 (35%)
2	5 (3%)	0 (0%)	9 (5%)	8 (1%)	22 (1%)
Primary lesion					
Resected	57 (31%)	241 (51%)	56 (29%)	147 (23%)	501 (34%)
Present	128 (69%)	233 (49%)	140 (71%)	490 (77%)	991 (66%)
Diagnosis					
Advanced	148 (80%)	396 (84%)	162 (83%)	527 (83%)	1233 (83%)
Relapse	37 (20%)	78 (16%)	34 (17%)	110 (17%)	259 (17%)
No. of metastatic sites					
≤1	86 (46%)	209 (44%)	88 (45%)	308 (48%)	691 (46%)
≥ 2	99 (54%)	265 (56%)	108 (55%)	329 (52%)	801 (54%)
Treatment regimen					
S‐1 alone	101 (55%)	239 (50%)	99 (51%)	‐	439 (29%)
S‐1 plus cisplatin	84 (45%)	‐	‐	321 (50%)	405 (27%)
S‐1 plus docetaxel	‐	235 (50%)	‐	‐	235 (16%)
S‐1 plus irinotecan	‐	‐	97 (49%)	‐	97 (7%)
S‐1 plus oxaliplatin	‐	‐	‐	316 (50%)	316 (21%)

Abbreviation: ECOG PS, Eastern Cooperative Oncology Group Performance Status.

The main results of each trial are summarized in Table 2. The median PFS period was comparable among the four trials, whereas the median OS and PPS periods were the longest in the G‐SOX trial. In total, 22, 650, 487, and 333 patients had best response of CR, PR, SD, and PD, respectively, accounting for 672 (45%) responders and 820 (55%) nonresponders. Among 1492 patients, 1190 (80%) received second‐line chemotherapy.

**TABLE 2:** Summary of trial results.

	Spirits *N* = 185	Start *N* = 474	GC0301/TOP‐002 *N* = 196	G‐SOX *N* = 637
Median OS (months, IQR)	11.7 (IQR 6.2–18.8)	11.3 (IQR 6.2–20.5)	11.0 (IQR 6.4–18.4)	14.4 (IQR 8.3–22.1)
Median PFS (months, IQR)	4.0 (IQR 2.2–6.9)	4.1 (IQR 2.1–7.0)	4.2 (IQR 2.3–6.1)	4.4 (IQR 2.8–7.7)
Median PPS (months, IQR)	6.2 (IQR 2.6–11.7)	6.0 (IQR 2.4–12.4)	6.6 (IQR 3.3–12.0)	9.1 (IQR 4.4–13.9)
Tumor response				
CR	2 (1%)	14 (3%)	0 (0%)	6 (1%)
PR	78 (42%)	149 (31%)	75 (38%)	348 (55%)
SD	47 (25%)	174 (37%)	74 (38%)	192 (30%)
PD	58 (31%)	137 (29%)	47 (24%)	91 (14%)
Responder	80 (43%)	163 (34%)	75 (38%)	354 (56%)
Non‐responder	105 (57%)	311 (66%)	121 (62%)	283 (44%)
Second‐line chemotherapy				
Received	148 (80%)	345 (73%)	170 (87%)	527 (83%)
Not received	37 (20%)	118 (25%)	26 (13%)	88 (14%)
Unknown	0 (0%)	11 (2%)	0 (0%)	22 (3%)

Abbreviation: CR, complete response; IQR, interquartile range; OS, overall survival; PD, progressive disease; PFS, progression‐free survival; PPS, postprogression survival; PR, partial response; SD, stable disease.

### 3.2 Individual‐level correlation among OS, PFS, and PPS

**FIGURE 2:**
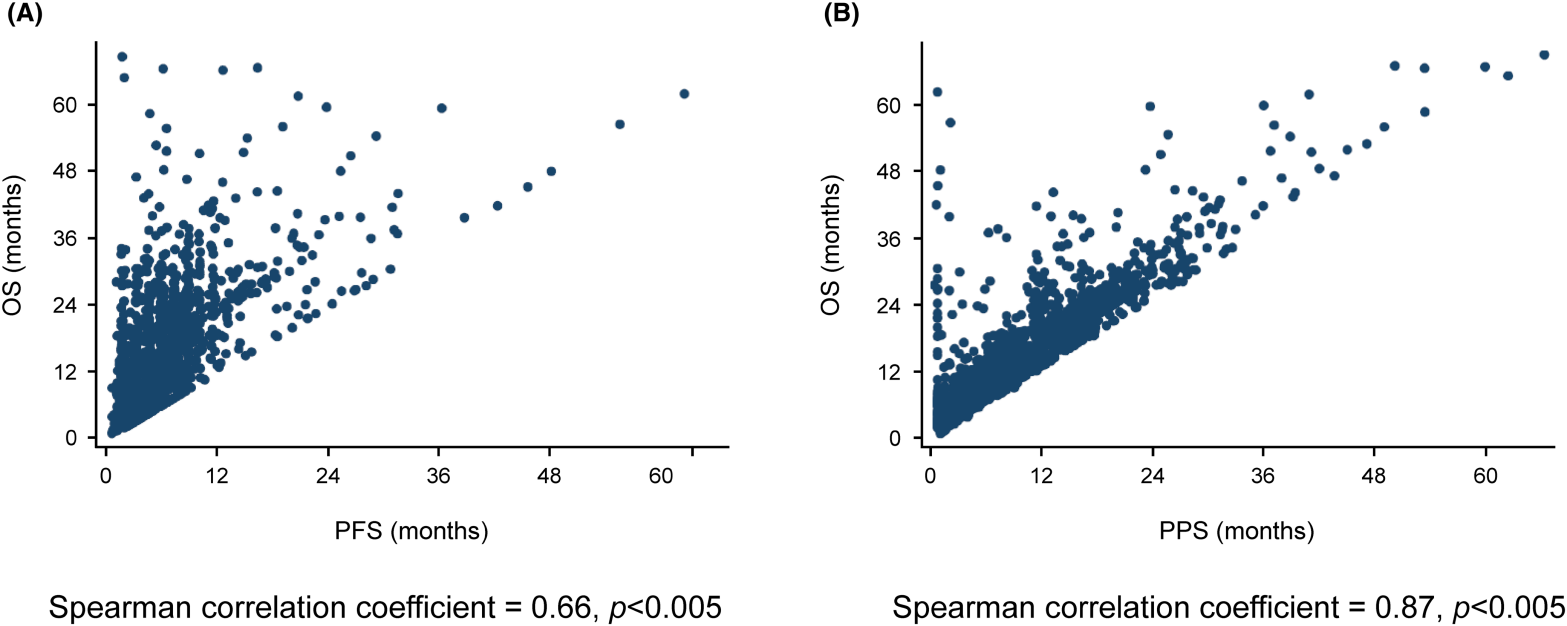
Correlation between survival outcome endpoints. (A) Correlation between PFS and OS. (B) Correlation between PPS and OS. OS, overall survival; PFS, progression‐free survival; PPS, postprogression survival.

The correlation of OS and PFS was moderate (Spearman correlation coefficient = 0.66, *p* < 0.005) and that of OS and PPS was strong (Spearman correlation coefficient = 0.87, *p* < 0.005) (Figure 2). The trend of correlations of OS‐PFS and OS‐PPS were similar between S‐1 alone and doublet regimen (Figure S1).

The correlation between PFS and PPS was weak (Spearman correlation coefficient = 0.23, *p* < 0.005) (Figure S2).

### 3.3 Individual‐level correlation between tumor response and other clinical outcomes

**FIGURE 3:**
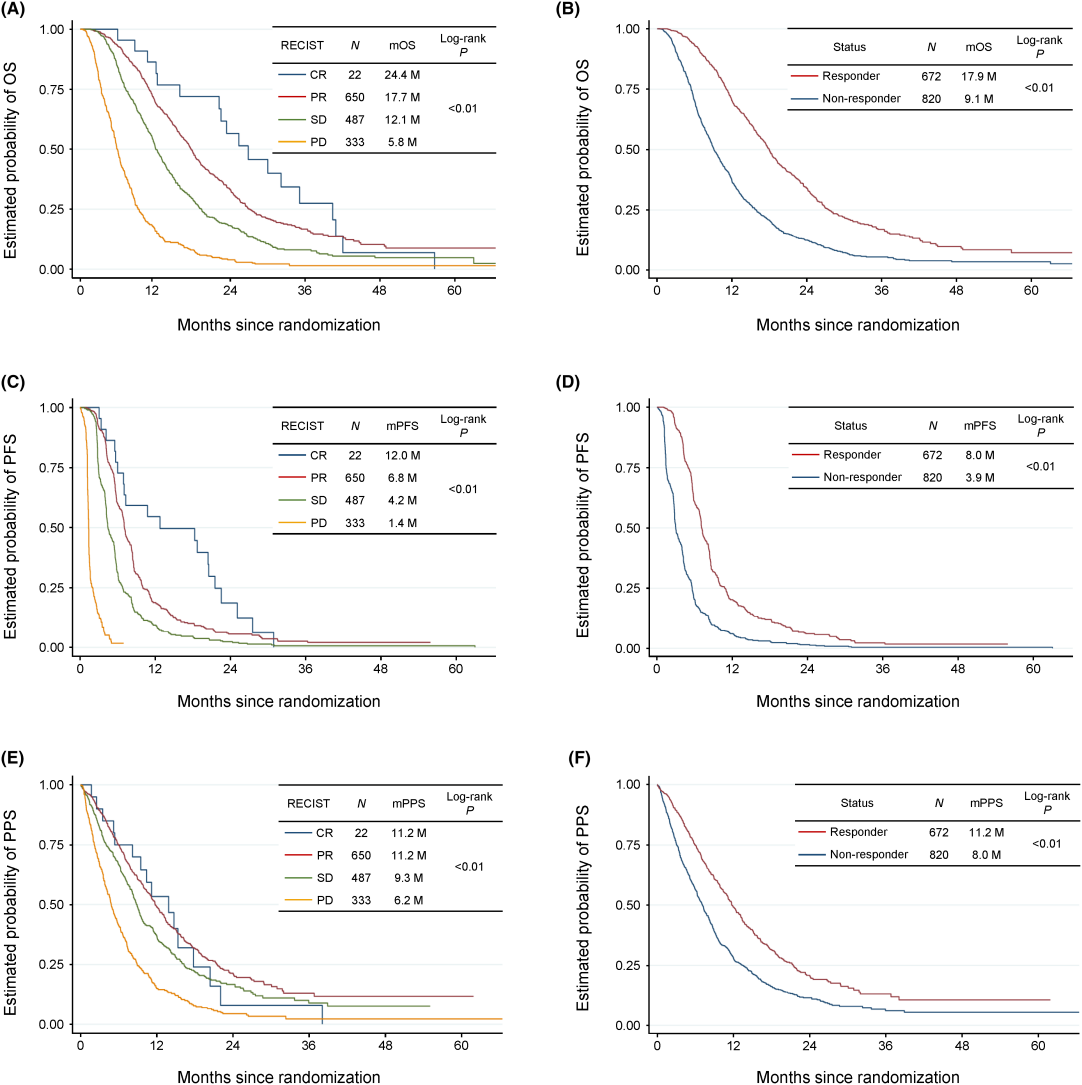
Survival outcomes compared between RECIST (Response Evaluation Criteria in Solid Tumors) categories. (A) Kaplan–Meier curves of OS in each RECIST category. (B) Kaplan–Meier curves of OS in responders and nonresponders. (C) Kaplan–Meier curves of PFS in each RECIST category. (D) Kaplan–Meier curves of PFS in responders and nonresponders. (E) Kaplan–Meier curves of PPS in each RECIST category. (F) Kaplan–Meier curves of PPS in responders and nonresponders. CR, complete response; OS, overall survival; PD, progressive disease; PFS, progression‐free survival; PPS, postprogression survival; PR, partial response; RECIST, Response Evaluation Criteria in Solid Tumors; SD, stable disease.

Patients with better RECIST categories had longer median OS, PFS, and PPS periods (longer in CR, PR, SD, and PD in this order). Compared with nonresponders, responders showed significantly longer OS (median, 17.7 vs. 9.1 months, *p* < 0.005), PFS (median, 6.9 vs. 2.8 months, *p* < 0.005), and PPS (median, 10.5 vs. 6.0 months, *p* < 0.005) periods (Figure 3).

Responders showed higher prevalence of ECOG PS 0 and one or less metastatic sites than nonresponders, whereas other patient characteristics were comparable between the groups (Table 3). The proportion of patients who received second‐line chemotherapy was significantly lower among responders (74% [498/672] vs. 84% [692/820], *p* < 0.005).

**TABLE 3:** Patient characteristics compared between responder and nonresponder groups.

	Responder *N* = 672		Nonresponder *N* = 820		*p*‐Value
Sex					
Male	501 (75%)		610 (74%)		0.94
Female	171 (25%)		210 (26%)		
Age					
Median (Range)	65 (28–83)		64 (21–85)		0.14
ECOG PS					
0	466 (69%)		480 (59%)		<0.01
1	198 (29%)		326 (40%)		
2	8 (1%)		14 (2%)		
Primary lesion					
Resected	214 (32%)		287 (35%)		0.20
Present	458 (68%)		533 (65%)		
Diagnosis					
Advanced	565	(84%)	668	(81%)	0.18
Relapse	107	(16%)	152	(19%)	
No. of metastatic sites					
≤ 1	335	(50%)	356	(43%)	0.01
≥ 2	337	(50%)	464	(57%)	

Abbreviation: ECOG PS, Eastern Cooperative Oncology Group Performance Status.

## 4 DISCUSSION

In this study, we comprehensively examined the correlation between multiple clinical endpoints at the individual level in patients with AGC who received first‐line chemotherapy. We reacknowledged that PFS is a mild surrogate for OS and its prognostic effect is possibly diluted by a strong correlation between PPS and OS. The positive correlation between tumor response and other all survival outcomes suggested that tumor response would be another individual‐level surrogate useful for patient management.

PFS is a well‐reported individual‐level surrogate in the first‐line chemotherapy of patients with AGC. A literature‐based study showed a moderate correlation between PFS and OS (Spearman correlation coefficient = 0.70), whereas an IPD‐based study showed a strong correlation (Spearman correlation coefficient = 0.853).^12^, ^19^ Compared with these previous results, the results of the present study presented weaker correlation between PFS and OS (Spearman correlation coefficient = 0.66). This discrepancy might be due to the difference in the frequency of subsequent chemotherapies between the studies. In general, second‐line chemotherapy is more prevalent in Japan than in Western countries.^20^ In this study, most patients received second‐line chemotherapy. Thus, greater availability of second‐line treatment might attenuate the prognostic effect of PFS that has been observed in a meta‐analysis of mainly international clinical trials.

Interestingly, we demonstrated a strong positive liner correlation between OS and PPS (Spearman correlation coefficient = 0.87). The result was consistent with that of a Japanese retrospective study showing a similar correlation between OS and PPS (Spearman correlation coefficient = 0.87) in patients with AGC who received first‐line fluoropyrimidine plus platinum.^14^ Our findings reindicate the importance of postprogression therapies after failure of first‐line treatment.^13^, ^21^

Although tumor response is a traditional clinical endpoint, it has not been well‐reported for surrogacy in the first‐line treatment of AGC. To the best of our knowledge, no IPD‐based meta‐analyses have investigated the correlation between tumor response and OS in the first‐line treatment of AGC. Only a literature‐based meta‐analysis showed a moderate correlation between the response rate and OS.^22^ However, it was conducted when standard first‐line regimens and availability of subsequent treatments were largely different from the current situation. This study demonstrated that responders had significantly better OS and PFS than nonresponders, with a trend for a longer survival period in better RECIST categories. PPS was also positively associated with tumor response. These results would be informative for patient management because tumor response can predict patient prognosis early. We can speculate two mechanisms for the significant correlation between tumor response and all survival outcomes. First, patients who experienced tumor response might harbor relatively less tumor burden and be in a better general condition at the time of progression during the first‐line treatment, leading to improved OS and PPS. Thus, tumor response could have direct effects on survival, representing its causal or potential surrogacy for OS. Second, confounding factors are present. In general, the same factors tend to be prognostic for both the surrogate and survival, creating an apparent correlation between these endpoints.^11^ Thus, known and unknown prognostic factors might induce positive correlation between tumor response and survival outcomes. In fact, responders had better ECOG PS and fewer metastatic sites. Whereas, the proportion of patients receiving second‐line chemotherapy was lower among responders. Although the specific reason regarding this point is unclear, the difference in rate of second‐line chemotherapy could affect the results of PPS and OS between responders versus nonresponders. However, no statistical method can determine how the results were mediated by the above mechanisms; thus, these explanations remain speculative.

This study had several limitations. First, trial‐level surrogacy was not investigated. To address if tumor response can be adequately used as a surrogate endpoint in clinical trials comparing ≥2 regimens, larger meta‐analyses are needed. Second, this pooled analysis only contained Japanese trials of S‐1 based chemotherapy that is not widely used in global. Thus, our findings should be carefully extrapolated into the clinical situation outside Japan. Finally, this study was conducted before the era where immune checkpoint inhibitors are introduced in combination with the standard first‐line treatment. Thus, the results may not reflect the current clinical situation. Several previous studies showed neither ORR nor PFS had apparent correlation to OS in patient with advanced solid tumors treated with immune checkpoint inhibitors.^23^, ^24^, ^25^ Further studies are warranted to validate our findings in AGC patients treated with first‐line chemotherapy containing immune checkpoint inhibitors.

In conclusion, this IPD‐based pooled analysis showed positive linear correlation between OS and PFS and between OS and PPS in patients with AGC treated with first‐line chemotherapy. Significant associations between RECIST categories and survival outcomes suggested that tumor response could be useful to predict individual‐level prognosis.

## AUTHOR CONTRIBUTIONS

**Hiroyuki Arai:** Conceptualization (lead); methodology (equal); visualization (equal); writing – original draft (lead). **Madoka Takeuchi:** Conceptualization (equal); data curation (lead); formal analysis (lead); methodology (equal); software (lead); visualization (equal); writing – review and editing (equal). **Wataru Ichikawa:** Investigation (equal); resources (equal); supervision (equal); writing – review and editing (equal). **Kohei Shitara:** Investigation (equal); resources (equal); writing – review and editing (equal). **Yu Sunakawa:** Investigation (equal); resources (equal); writing – review and editing (equal). **koji oba:** Investigation (equal); resources (equal); writing – review and editing (equal). **Wasaburo Koizumi:** Investigation (equal); resources (equal); writing – review and editing (equal). **Yuh Sakata:** Investigation (equal); resources (equal); writing – review and editing (equal). **Hiroshi Furukawa:** Investigation (equal); resources (equal); writing – review and editing (equal). **Yasuhide Yamada:** Investigation (equal); resources (equal); writing – review and editing (equal). **Masahiro Takeuchi:** Investigation (equal); resources (equal); writing – review and editing (equal). **Masashi Fujii:** Project administration (lead); supervision (lead); writing – review and editing (equal).

## FUNDING INFORMATION

This work was supported by Taiho Pharmaceutical Co., Ltd, Yakult Honsha Co., Ltd, and Daiichi Sankyo Co., Ltd.

## CONFLICT OF INTEREST STATEMENT

WI reports honoraria from Chugai Pharma, AstraZeneca, Nihonkayaku, Merck Serono, Bristol‐Myers Squibb Japan, Yakult Honsha, Taiho Pharmaceutical, Bayer Yakuhin, and Kyowa Kirin; KS reports research funding from Astellas Pharma, Ono Pharmaceutical, Daiichi Sankyo, Taiho Pharmaceutical, Chugai Pharma, MSD, Amgen, Eisai, and Medi Science, advisory role for Eli Lilly and Company, Bristol‐Myers Squibb, Takeda Pharmaceuticals, Pfizer Inc, Ono Pharmaceutical, Novartis, AbbVie Inc, Daiichi Sankyo, Taiho Pharmaceutical, GlaxoSmithKline, Amgen, Boehringer Ingelheim, MSD, Astellas, Guardant Health Japan, and Janssen, and honoraria from Bristol‐Myers Squibb, Takeda Pharmaceuticals, and Janssen; YS1 reports grants/contracts from Chugai Pharmaceutical, Taiho Pharmaceutical, Takeda, Sanofi, Ohtsuka Paharmaceutical, and Eli Lilly Japan, honoraria from Eli Lilly Japan, Bristol‐Byers Squibb, Chugai Pharmaceutical, Takeda, Ono Pharmaceutical, Merck Biopharma, Taiho Pharmaceutical, Bayer, Daiichi‐Sankyo, MSD, Sysmex, and Guardant Health, and advisory board for Merck Biopharma, Ono Pharmaceutical, and Guardant Health; KO reports honoraria from Chugai pharmaceutical company and data safety monitoring board/advisory board for ONO pharmaceutical company, Janssen pharmaceutical company, and AsahiKasei pharmaceutical company; YS2 reports honoraria from Taiho Pharmaceutical Co., Ltd. and Yakult Honsya Co., Ltd.; YY reports honoraria from Taiho, Behringer‐Ingelheim, and Ono. All remaining authors have declared no conflicts of interest.

## ETHICS APPROVAL AND CONSENT TO PARTICIPATE

All clinical trials were approved by the respective competent ethical committees according to the Declaration of Helsinki and Japanese Ethical Guidelines for Medical and Health Research Involving Human Subjects.

## Supporting information


Figure S1:

Figure S2:

Table S1.

## Data Availability

The data used to support the findings of this study are available from the corresponding author upon request.
