# Supplementary material for: Correlation of multiple endpoints in the first‐line chemotherapy of advanced gastric cancer: Pooled analysis of individual patient data from Japanese Phase III trials
Source: Cancer Med. 2023 Dec 23;13(1):e6818. doi: 10.1002/cam4.6818 (PMC10807593; doi:10.1002/cam4.6818)
Supplement: Supplementary file 1 — Figure S1: [file CAM4-13-e6818-s001.pdf]

Figure S1

Correlation of OS and PFS
in patients receiving S-1 alone

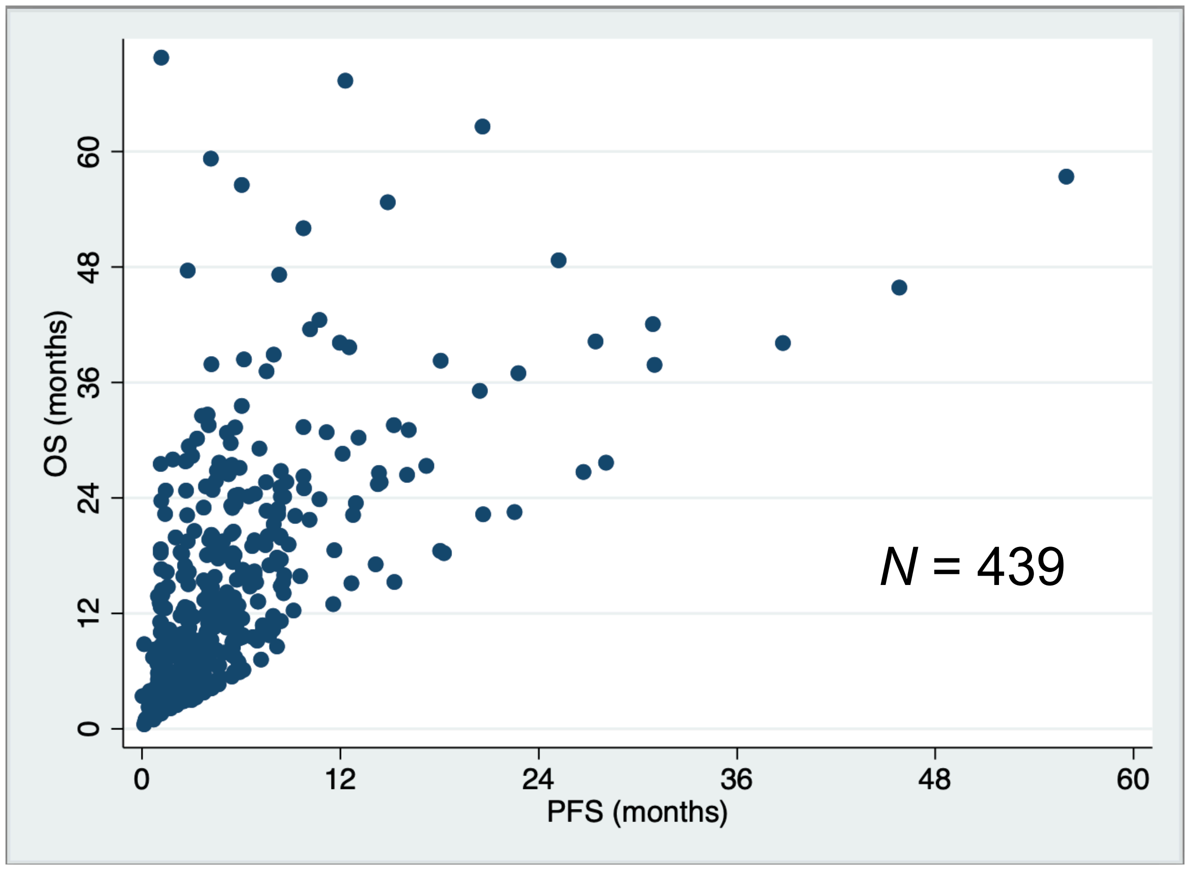

Spearman correlation coefficient = 0.67, $p<0.005$

Correlation of OS and PPS
in patients receiving S-1 alone

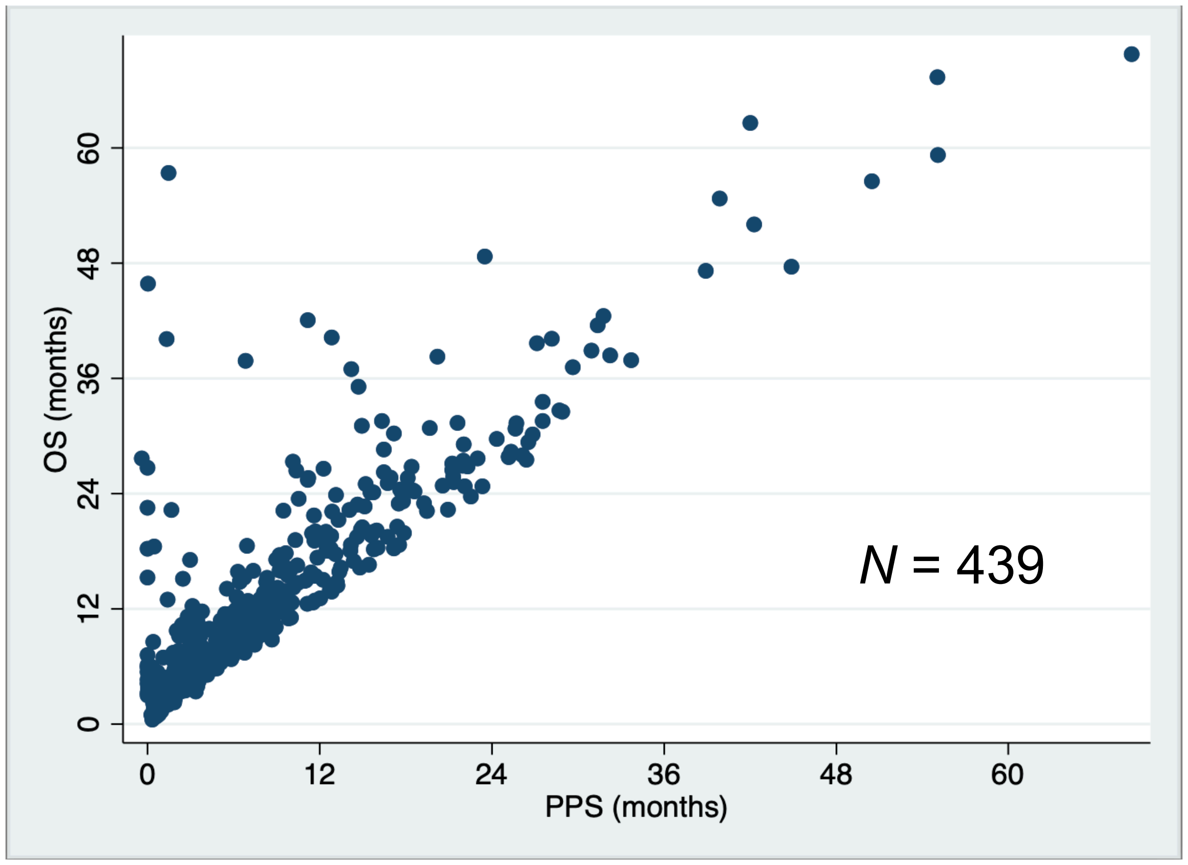

Spearman correlation coefficient = 0.84, $p<0.005$

Correlation of OS and PFS
in patients receiving S-1+α (doublet regimen)

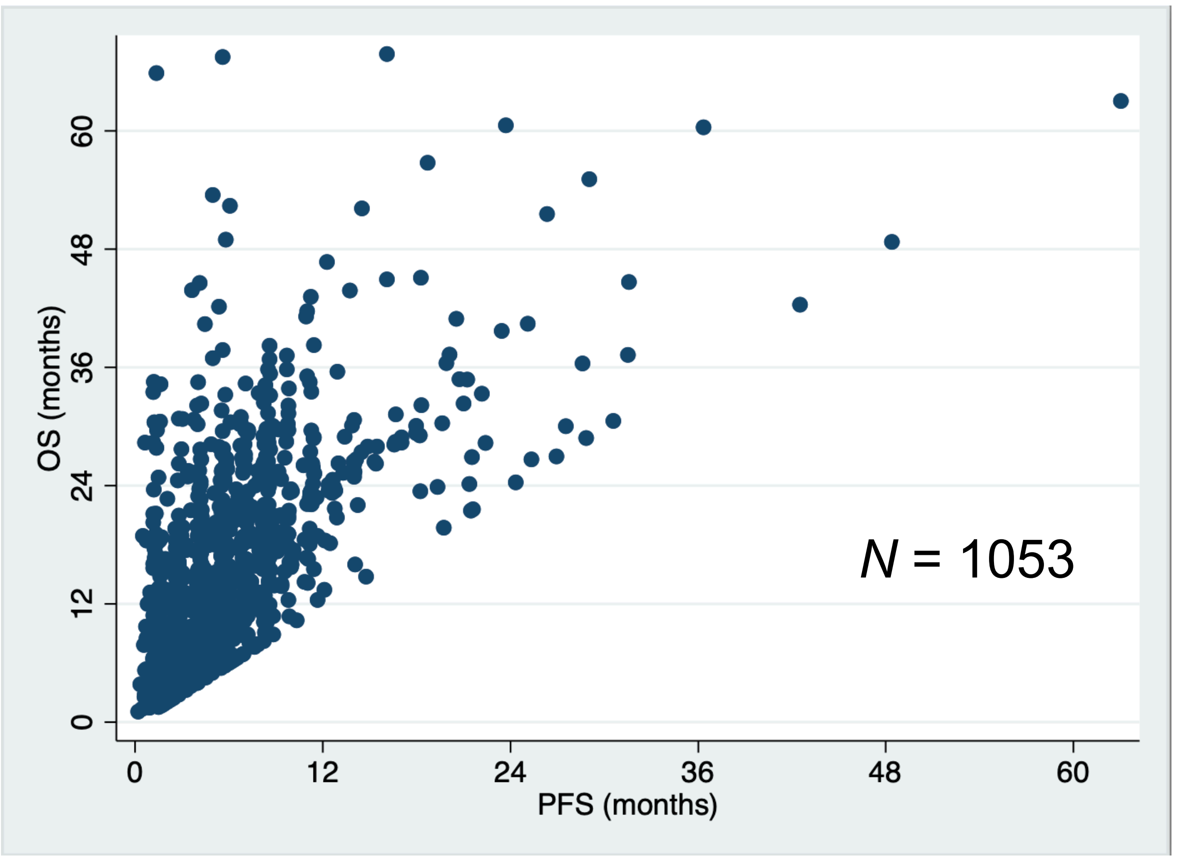

Spearman correlation coefficient = 0.65, $p<0.005$

Correlation of OS and PPS
in patients receiving S-1+α (doublet regimen)

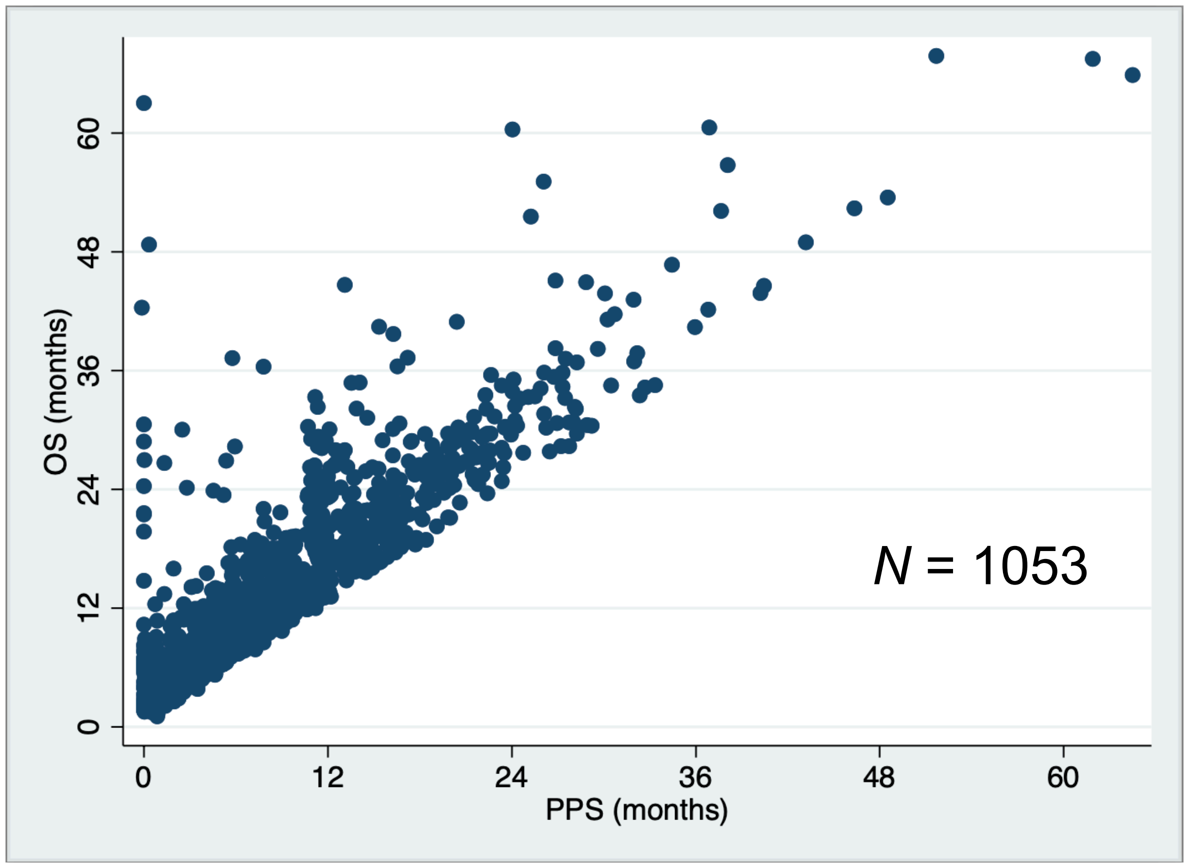

Spearman correlation coefficient = 0.87, $p<0.005$
