# Supplementary figures and images for: Correlation of multiple endpoints in the first‐line chemotherapy of advanced gastric cancer: Pooled analysis of individual patient data from Japanese Phase III trials
Source: Cancer Med. 2023 Dec 23;13(1):e6818. doi: 10.1002/cam4.6818 (PMC10807593; doi:10.1002/cam4.6818)

**Figure S2**

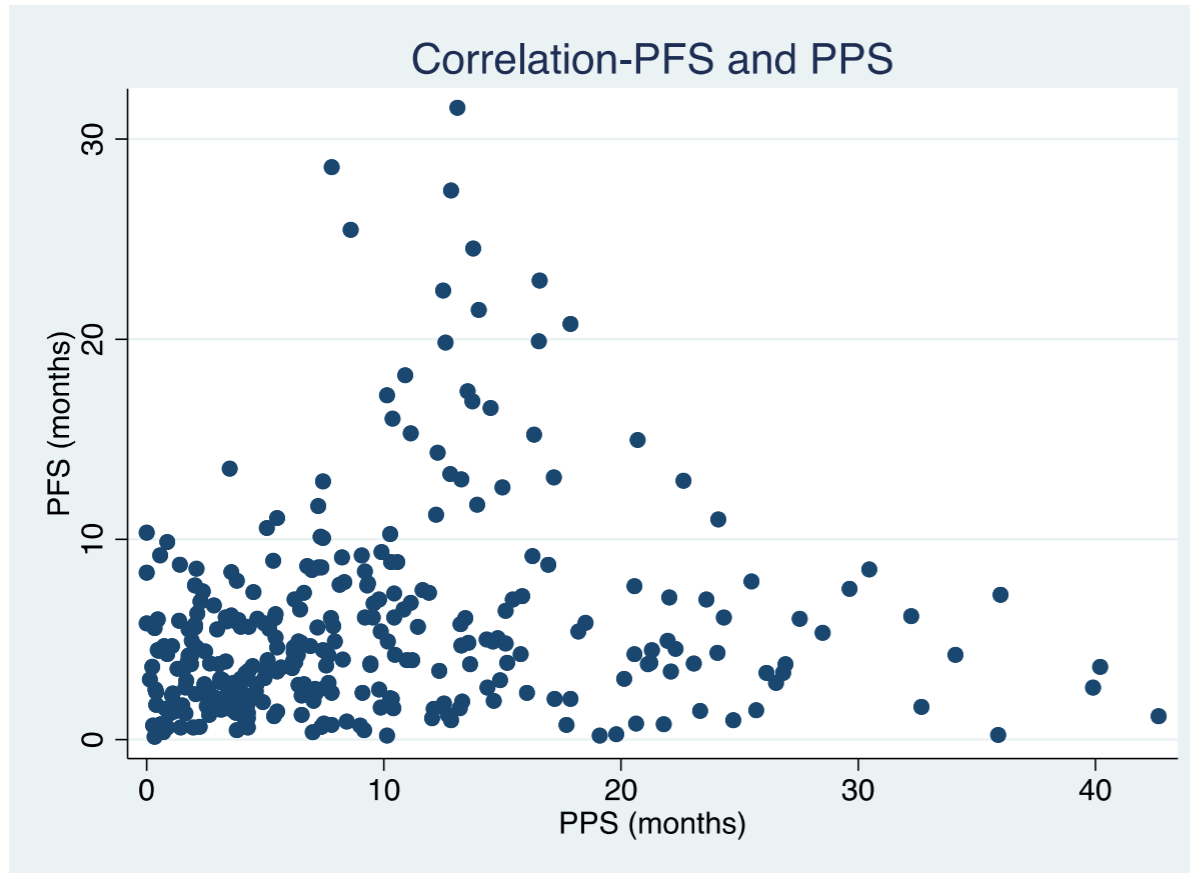

Spearman correlation coefficient = 0.23,  $p < 0.005$

Supplement: Supplementary file 2 — Figure S2: [file CAM4-13-e6818-s003.pdf]
