# Supplementary material for: Correlation of multiple endpoints in the first‐line chemotherapy of advanced gastric cancer: Pooled analysis of individual patient data from Japanese Phase III trials
Source: Cancer Med. 2023 Dec 23;13(1):e6818. doi: 10.1002/cam4.6818 (PMC10807593; doi:10.1002/cam4.6818)
Supplement: Supplementary file 3 — Table S1. [file CAM4-13-e6818-s002.docx]

**Table S1. Interval of CT scan for RECIST assessment in each trial**

|  | SPIRITS | START | GC0301/TOP-002 | G-SOX |
| --- | --- | --- | --- | --- |
| Interval of CT scan | 2 months | 6 weeks | 6 weeks after treatment start and thereafter every 2 months | 6 weeks |

Abbreviation: RECIST, Response Evaluation Criteria in Solid Tumors.
